# Salt for the Human System

**Published:** 1885-01

**Authors:** 


### SALT FOR THE HUMAN SYSTEM.

The London *Lancet* combats the folly of some would-be improvers on Galen, who decry the use of salt as a food condiment because it is a mineral. The *Lancet* says that common salt, chloride of sodium, is the most widely distributed substance in the body ; it exists in every fluid and in every solid ; and not only is everywhere present, but in almost every part it constitutes the largest portion of the ash when any tissue is burnt. In particular it is a constant constituent of the blood, and it maintains in it a proportion that is almost wholly independent of the quantity that is consumed with the food. The blood will take up so much and no more, however much we may take with our food, and, on the other hand, if none be given, the blood parts with its natural quantity slowly and unwillingly. Nothing can demonstrate its value better than the fact if albumen without salt is introduced into the intestines of an animal, no portion of it is absorbed, while it all quickly disappears if salt be added. The conclusion therefore is obvious that salt, being wholesome, and indeed necessary, should be taken in moderate quantities, and that abstention from it is likely to be injurious.

